# Key action areas for transforming the UK food system: insights from the Transforming UK Food Systems (TUKFS) Programme project portfolio

**DOI:** 10.1098/rstb.2024.0166

**Published:** 2025-09-18

**Authors:** Sarah Bridle, Kelly Parsons, Guy Poppy, Tracey Duncombe, Lynn V. Dicks, Bob Doherty, Alex Johnstone, Christian Reynolds, Carol Wagstaff, Fergus Lyon, Sam Buckton, Ben Dare, Martin White, Christopher Yap, Roya Shahrokni, Riaz Bhunnoo, Hannah Mitchell, Ioan Fazey, Dominic Moran, Christopher Turner, Jonathan Beacham, John Ingram, Peter Jackson, Rebecca Wells, Katherine Denby, Tom Macmillan, Jeffrey M. Brunstrom, Maria Bryant

**Affiliations:** ^1^ Department of Environment and Geography, University of York, York YO10 5NG, UK; ^2^ MRC Epidemiology Unit, School of Clinical Medicine, University of Cambridge, Cambridge CB2 0QQ, UK; ^3^ University of Bristol, Bristol BS8 1QU, UK; ^4^ Department of Food and Nutritional Sciences, University of Reading, Reading RG6 6DZ, UK; ^5^ Department of Zoology, University of Cambridge, Cambridge CB2 3EJ, UK; ^6^ School for Business and Society, University of York, York YO10 5DD, UK; ^7^ The Rowett Institute, University of Aberdeen, Aberdeen AB25 2ZD, UK; ^8^ Centre for Food Policy, City St George’s, University of London, London EC1V 0HB, UK; ^9^ Centre for Enterprise, Environment and Development Research, Middlesex University London, London NW4 4BT, UK; ^10^ Unaffiliated; ^11^ Global Food Security Programme, UKRI, Swindon SN2 1UH, UK; ^12^ Institute of Geography, University of Edinburgh, Edinburgh, Scotland, UK; ^13^ University of Greenwich, London, UK; ^14^ Business School, University of Bristol, Bristol BS8 1SD, UK; ^15^ Environmental Change Institute, University of Oxford, Oxford OX1 3QY, UK; ^16^ Department of Geography, University of Sheffield, Sheffield S10 2TN, UK; ^17^ Department of Biology, University of York, York YO10 5DD, UK; ^18^ Royal Agricultural University, Cirencester GL7 6JS, UK; ^19^ National Institute for Health and Care Research Bristol Biomedical Research Centre and the National Institute for Health and Care Research Applied Research Collaboration West (NIHR ARC West), Bristol, UK

**Keywords:** food system, regenerative agriculture, food manufacturing, food environment, food community, food policy

## Abstract

The UK food system is a driver of the public health crisis of non-communicable disease, is linked to the cost-of-living crisis, and contributes to climate change, biodiversity loss and soil degradation. The economy relies strongly on the health of its people and food businesses, while also impacting the livelihoods of food system actors. However, action towards more resilient, equitable and regenerative food systems remains too slow and unambitious to adequately address these challenges. The Transforming UK Food Systems Programme comprises a wide range of research projects which address these challenges in a novel place-based, co-produced and action-oriented way. We provide 27 suggested action areas for supporting food system transformation, grouped in five themes spanning production, manufacturing, supply chain and consumption. Among the suggestions, there is a strong emphasis on the importance of co-production with food system actors and affected citizens. We highlight the vital role of governance and policy in supporting these action areas in both a structural and financial way, noting that this needs both national policy and regional approaches to take into account geographically varying cultural circumstances and values, and to allow the high level of co-production necessary.

This article is part of the theme issue ‘Transforming terrestrial food systems for human and planetary health’.

## 1. Introduction

The food system is estimated to contribute between one quarter and one third of all current greenhouse gas emissions [1,2], and this sector alone is on track to cause over 1.5°C of global warming by 2100 [3]. Furthermore, the food system is the lead cause of major environmental degradation worldwide—including biodiversity loss, deforestation and freshwater pollution and depletion [4]. Meanwhile, poor diet is estimated to be a cause of 22% of adult deaths globally, particularly due to increasing levels of non-communicable diseases, including cardiovascular disease and type 2 diabetes [5].

The food system is shaped by the decisions of stakeholders involved in production through to consumption, and aside from a food safety agenda, there has been little oversight or overall control beyond economic and political interests [6]. As a consequence, the system is not moving towards the UN Sustainable Development Goals [7] to end hunger and malnutrition, with billions of people lacking access to nutritious, safe and sufficient food [8]. The consequences for the health of the planet and its people have been branded a global ‘syndemic’ and have led to calls for major dietary change (e.g. [9]) and transformation of the food system [6,10,11].

In this article we focus on the UK, in which 15% of adult deaths are attributed to diet [5] and where 26−30% of greenhouse gas emissions come from the UK food system [12,13]—12% of UK emissions are from agriculture [14] with the remainder of food system emissions coming from non-agriculture parts. At the same time, 2.5 million adults (5% of all households) reported not eating for a whole day due to lack of access to food, and 18% of households with children experienced food insecurity in the previous month [15]. Calls to account for the resulting externalized costs (e.g. to health services) run into the challenge of providing affordable food given increasing levels of household food insecurity [16].

To provide and assess the evidence for ways to address these challenges in the UK food system, the Transforming UK Food Systems (TUKFS) Programme was created through a partnership spanning UK Research and Innovation (UKRI) and UK government departments, supported through a major investment (£47.5 million) by the UKRI Strategic Priorities Fund [17]. For more detail on the programme, including its rationale and research methodologies, see the introductory article to this issue [18]; the findings from the TUKFS Programme are the main focus chapters in this Special Issue.

This article draws together action areas from across the TUKFS Programme, grouped under five themes that arose from a workshop held by the TUKFS Programme: (A) disrupting towards a regenerative food production system; (B) innovating in manufacturing and the supply chain; (C) transforming food environments for healthy and sustainable food; (D) empowering communities; and (E) transforming policy and governance. However, we note that a systems approach to transformation is essential because all the themes are interconnected [17]. We also note there are some action areas that fall under multiple headings, and that policy and governance *recommendations* run across themes, but the final theme predominantly discusses *research on* policy and governance. Our aim is to summarize the work of the TUKFS Programme—we therefore draw out action areas from work across the programme, rather than reviewing the wider evidence base on actions to transform the food system.

## 2. Theme A: disrupting towards a regenerative food production system

Ideas and practices related to regenerative systems are gaining traction in both academic and non-academic fields, particularly in agriculture [19,20]. Regenerative approaches are underpinned by more holistic and mutualistic relations between people and wider nature. For example, a fully regenerative farm would not only employ practices that advance the farmer’s economic needs, and regenerate soils and ecosystems on which the farm directly depends, but would also regenerate wider social and ecological environments—e.g. by boosting pollinator populations and acting as a hub for community interaction [19]. The Regenerative Lens framework [19] identifies key qualities of regenerative systems (e.g. food systems); for example, in a regenerative food system, diversity is increased on all levels from multi-cropping to procurement [21,22], cooperation and reciprocity along the value chain foster common goals [23], and peer-to-peer learning communities embed context-dependent solutions. Conceptually, regenerative food systems can be considered to go beyond simply reducing anthropogenic harm to acceptable levels, instead moving to actively ‘spiralling up’ (i.e. continually and iteratively accelerating) beneficial social and ecological health outcomes. For example, in terms of climate change mitigation, a regenerative food system implies a shift from a ‘net zero’ to a ‘carbon negative’ system.

The context-dependent and multi-scalar nature of food systems make it necessary for researchers to work across multiple scales, with diverse stakeholders embedded in places, to identify transformational or even disruptive actions. Thus, Buckton *et al.* [24], working with multiple stakeholders from different parts of the food system (114 in total), developed a set of potential actions expected to enable transformation to a regenerative food system, at the level of a region—Yorkshire, UK.

Actions must be feasible, including taking into account acceptability to those who might consider themselves to be impacted by the changes. Two examples illustrate why this is important. First, the development of new technologies to reduce greenhouse gas emissions in the human food chain can encounter unexpected but serious issues of public acceptance, for example around genetic modification. Second, novel food sources, such as land-based marine seafood production systems [25], have implications that can seem positive or negative to different stakeholder groups, even while helping reduce greenhouse gas emissions. Research with UK farmers on the topic of cultured meat found generalized concern that the technology will drive corporate consolidation and threaten rural livelihoods, yet discussions with farmers about their specific businesses led to a nuanced engagement with the potential pros and cons of the technology [26,27].

Transforming food production systems is likely to require profound systemic changes to values [28]. Conflicting worldviews and values among stakeholder groups can create inaction, leading to inertia. In this context, framing messages in ways that resonate with the beliefs and attitudes of actors can increase motivation for change [29]. We therefore suggest working with different stakeholders and *convening dialogues to reconcile conflicting views* through consensus-building where possible.

Another promising approach is for researchers to study actions that are already emerging from the food system and appear to have potential to be transformative or disruptive. ‘Regenerative agriculture’ is an example of this that is widely regarded as a grassroots, farmer-led movement that has gathered substantial momentum in many parts of the world, in private and public sectors [21]. Beacham *et al.* [30] demonstrated its emotional appeal from farmer perspectives. However, thus far there is a limited evidence base and its definition is contested (for a discussion of lessons learned see Berthon *et al*. [21]).

Commonly, regenerative agriculture involves applying a set of five principles: (i) reduce soil disturbance, (ii) increase crop diversity, (iii) keep the soil covered, (iv) keep living roots all year round, and (v) introduce livestock [21,31]. These principles guide the choice of farming practices in a context-dependent way, aiming to achieve a variety of beneficial economic, social and environmental outcomes. A UK-wide farmer survey [31] showed that while mixed and arable farmers had high levels of awareness (>60%) and moderate uptake of sustainable soil management practices (>30%), these practices were not always being combined in ways that correspond to the full set of regenerative agriculture principles.

With a few exceptions (e.g. [32,33]), the majority of research measuring outcomes of regenerative agriculture practices has focused on impacts of single agricultural practices, such as cover crops (e.g. [34]) or reduced tillage (e.g. [35]), and on single or few outcomes, rather than considering whole system change involving multiple practices, overarching principles and complex outcomes.

Furthermore, there is an uncertain and complicated policy environment and geopolitical landscape around the transition to regenerative agriculture, in which farmers gather information from a fragmented range of sources, including publications, the press, social media and their own neighbours [30]. We suggest *supporting the development of independent agronomic advice on regenerative agriculture, peer-to-peer learning among farmers and a network of demonstration, or ‘lighthouse’, farms*. These are real world farms that demonstrate and implement sustainable practices [23].

Conducting research on emerging system change in any part of the food system demands transdisciplinary approaches to co-design, in which researchers work directly with food system actors from the outset, to ensure the research design is relevant and realistic in its choice of combined actions and monitored outcomes [29]. The TUKFS Programme has focused on regenerative agriculture through two complementary approaches at different scales that adhere to these principles [23,36].

The timescale for the benefits of regenerative agricultural practices are not well matched to the duration of the most common leasehold agreements, or research funding cycles. It takes years to rebuild soil structure after years of intensive agriculture, and some farmers report lower yields during the transition period [23]. Regenerative systems can be based on a 10 year rotation, involving a range of crops and practices, and soil carbon storage rates need to be measured on this timescale to assess impacts on climate change. In these circumstances, even a well-designed four-year experiment provides limited insight into the long-term economic and environmental impacts of the transition [21]. It is therefore necessary to *fund long-term (10 years or more) research on regenerative agriculture, co-designed with farmers, to provide salient evidence on environmental outcomes and financial viability*. The TUKFS trial plots have secured funding for several more years from both UK Government and from charitable foundations interested in more sustainable approaches to food systems.

**Summary of action areas^1^ on Theme A: disrupting towards a regenerative food production system**

A1. Co-produce messaging about changes to food production, to take into account the concerns of affected stakeholders [21,23,26,27,29]. Action: Defra, National Farmers Union, farmer groups, e.g. Future Farmers [23,36], and manufacturers and processors purposely procuring regenerative produce.

A2. Support the development of independent agronomic advice on regenerative agriculture, peer-to-peer learning among farmers and a network of demonstration, or ‘lighthouse’, farms [30]. Action: Defra^2^, research evidence generators, e.g. Rothamsted and AHDB, networks connecting research evidence generators and farming groups, e.g. the AFN Network+ [37].

A3. Fund long-term (10 years or more) research on regenerative agriculture, co-designed with farmers, to provide salient evidence on environmental outcomes and financial viability [36]. Action: Research funders and Defra.

## 3. Theme B: innovating in manufacturing and the supply chain

The food and drink manufacturing sector is the largest manufacturing sector in the UK [38]. The triple challenge for this sector is to improve food security, have resilience in supply chains and environmental sustainability [39]. Innovation is key to ensure food security and healthier nutrition for a growing UK population; to support the livelihoods of those people working in the food supply chain; to contribute substantially to the UK economy; and to do so in an environmentally sustainable way (healthier, greener and fairer). Maintaining the economic profitability of this sector allows companies to stay competitive in a global market, by adapting to changing consumer tastes, developing new products to meet evolving trends, improving operational efficiency and addressing sustainability concerns, ultimately enabling growth and market share expansion. Food manufacturing innovation can contribute to achieving the global and UK environmental sustainability goals, including reduced CO_2_ emissions, reduced food waste, more effective food distribution and increased recycling of plastic packaging. However, food manufacturing contributes significantly to current health and environmental externalities, so there is an underlying tension between the system’s economic and health and environmental goals.

The ‘supply chain’ is part of the food system, which refers to the movement and transformation of food from production to the consumer. It involves a network of companies, facilities and activities involved in sourcing, manufacturing and delivery of products, encompassing everything from raw materials to finished goods reaching the consumer. The UK is deeply integrated into global supply chains, due to its significant import and export activity. This is especially true for fresh produce like fruit and vegetables, where the UK food system is highly dependent on food imports from climate vulnerable countries. Recent challenges to the supply chain include the disruptions caused by the global COVID pandemic where lockdowns disrupted manufacturing and transportation to cause in-store shortages for consumers. Brexit has also created supply chain disruptions, primarily due to the reintroduction of customs duties, non-tariff barriers and increased administrative costs for businesses trading with the EU. This has led to delays, increased costs and a need for businesses to adapt [16]. In spite of these challenges, the UK has established itself at the forefront of innovation, with high quality produce and technological advances combining to cater to the demands of consumers.

Shifting towards shorter domestic food supply chains (the sale of food closer to its production) may be an opportunity to increase the profitability of UK farm businesses. We propose *incentivization of production and consumption of UK-grown pulses and legumes*, which can help reduce the climate impact of diets when combined with reduced consumption of high emissions items including meat. For example, the incorporation of beans as flour into sliced white bread is a promising way to increase the nutritional properties (fibre and protein) of a commonly consumed staple food, given improvements in manufacturing processes [40]. The work of, ‘Raising the Pulse’ highlights the role of faba beans to improve the environmental, nutritional and health benefits of pulse-enhanced foods. In addition, uptake of new varieties of bean suitable for UK growing conditions can be increased by actions right across the supply chain from (i) community enterprise, to (ii) small business entrepreneurs, and (iii) adoption by food giants [41,42]. These concepts are described in more detail within this issue [42], where the ‘fork to farm’ paradigm has been applied to assess how UK grown navy beans can help transform the food system, by improving diet quality and environmental sustainability. This approach is an intentional disruption of the generally-used ‘farm to fork’ productionist paradigm so as to emphasize the importance of thinking about the value chain from the perspective of consumption. This also creates opportunities to change population-level diet and health outcomes, with a focus on reformulation of food and beverage products or new products for reduced fat, more dietary fibre, less salt and sugar, and fewer calories, as less than 0.1% of UK consumers meet current healthy eating recommendations [43]. Further, using an example combining institutional catering and home-cooking by using healthier ingredients, new public procurement practices and novel local production methods, the work exemplifies the need for ‘systemic innovation’ (i.e. coordinated adaptation across food system activities) so as to transform food system outcomes [42].

The advancement of controlled environment agriculture and vertical farming is driving innovation in UK manufacturing and supply chains by redefining local food production, enhancing sustainability and reducing reliance on imports [44]. Controlled environment agriculture offers the potential to grow food on less land, and with more efficient fertilizer use and closer geographical proximity to consumers [45], which can also reduce reliance on air-freighted products thus reducing climate impacts. Commercial vertical farms can use sophisticated AI within the growing tower to apply targeted light spectrums and growing conditions for improved nutritional yield. This involves the integration of a circular model of food production, where there is minimal waste and nutrients are continually recycled, to embrace a low-emissions system. These systems embrace innovation in technology as part of a farming solution, where the economic viability to be part of a local food system is a key part of the evaluation [46,47].

Beyond its impact on manufacturing and supply chains, smaller-scale vertical farming can also play a vital role in fostering community engagement and education. Similar to the use of aquaponics in schools—which has been shown to enhance hands-on learning about healthy eating and environmental responsibility [48]—vertical farms can serve as living laboratories that engage local communities, schools and policymakers in discussions about future food systems [47]. There is significant research into optimizing growing conditions for plants as a food source with consideration of cost analysis, but much less into the socio-economic aspects [49]. We suggest *further research be undertaken to understand the conditions in which controlled environment agriculture can contribute to socio-economic outcomes* to tackle inequalities within the current food system, in terms of health and the environment [25,47].

Community-led action research can lead to social innovation, resulting in shorter supply chains and more direct access to local produce, e.g. local fish for local residents [50]. Co-design with local residents can increase uptake of low value and underutilized products, e.g. pouting, dogfish and whiting caught by local small vessels being delivered into local school meals. This can simultaneously provide health benefits, reduce waste and environmental damage (including climate impacts), and improve livelihoods by giving a fair price to local producers. Access to local food can also be developed through food hubs [51] and local delivery services, as well as using existing supermarkets who support small-scale producers [52]. Furthermore, enhancing local supply chains can improve food production resilience [53]. We suggest *supporting place-based supply chain innovation and social enterprise, incorporating community action to increase effectiveness and uptake*.

Innovation in supply chains can also improve the provision of UK food aid. In this context, food aid is an umbrella term used to describe any type of aid-giving activity which aims to provide relief from the symptoms of food insecurity and poverty and can describe a broad spectrum of activities, from small to large scale, local to national, emergency one-off operations or well-established food banks. A recent analysis of semi-structured interviews with 32 senior managers and experts from both commercial and food aid supply chains has led to detailed recommendations for a suite of actions to be made by surplus food donors, redistributors and governments [54]. The overriding conclusion is that *the UK Government needs to move from statutory guidance to legislative interventions on surplus or waste food to better serve disadvantaged groups (as in Italy, the US and France), with mandatory redistribution*. However, we note that using surplus as food aid is a short-term fix and does not address the underlying issue of poverty. Furthermore, we propose *increasing the role of logistics companies connecting commercial and food aid supply chains* to simultaneously reduce waste and ensure fresher, more sustainable food options for communities [54].

Food aid supply chains are already under great strain, and are particularly at risk from interruptions to food supply. However, interruptions can also create greater volumes of waste—e.g. the COVID-19 pandemic caused significant disruptions in the UK food services as nationwide stockouts led to unprecedented discrepancies between retail and home-delivery supply capacity and demand [55]. There is limited research into digital technology use in supply chains for the purpose of healthy food access, but a new framework proposes better food supply chain integration for urban food systems, incorporating principles from the Internet of Things [56]. We suggest *exploring emerging digital platforms as an innovative instrument in food supply chains*, to increase resilience in severe market disruptions and to increase healthy food access.

A challenge recognized in the independent review of the National Food Strategy was that responsibility for elements of food policy is split across multiple government departments. It identified the need for clear, long-term targets, ongoing political attention and a joined-up approach not only within government, but across the food industry and communities.

**Summary of action areas on Theme B: innovating in manufacturing and the supply chain**

B1. Research the conditions in which controlled environment agriculture can contribute to socio-economic outcomes [25,47]. Action: Research funders and research evidence generators to work in partnership with civil society organizations amplifying the voices of marginalized groups.

B2. Incentivize production and consumption of UK pulses [40–42]. Action: Defra, in partnership with growers.

B3. Support place-based supply chain innovation and social enterprise, incorporating community action to increase effectiveness and uptake [50]. Action: Defra and local government.

B4. Move from statutory guidance to legislative interventions on surplus or waste food to better serve disadvantaged groups, with mandatory redistribution [54]. Action: Government (local and national level).

B5. Increase the role of logistics companies connecting commercial and food aid supply chains [54]. Action: Defra and local government.

B6. Explore emerging digital platforms as an innovative instrument in food supply chains [55,56]. Action: Research funders and decision-makers in farmers and growers, decision-makers in processing and manufacturing, distribution and retail.

## 4. Theme C: transforming food environments for healthy and sustainable food

Food environments are considered to be a key interface within the food system as the point where food acquisition and consumption occurs as part of daily life [57,58]. Transforming food environments for healthier diets, nutrition and health is a foundational cornerstone of wider food system change. Food environments span built, cultivated, wild, social and digital food sources, as well as advertising which affects decision-making [57,59–61], providing multiple leverage points for interventions and action [62].

UK food environments are rapidly changing since the 2020 COVID-19 pandemic: there are more places to access food than ever before, and how we purchase and consume food is changing and diversifying through digitalization. This diversity can be seen through the results of a recent Food Standards Agency survey [63]: although 75% of UK respondents (*n* = 5812) reported that they bought food from a large supermarket about once a week or more often, there is also evidence of flourishing alternative and online food sectors. In the same survey, 44% of UK respondents had bought food from local/farmer’s markets or farm shops two to three times a month; 60% of respondents reported that they had ordered food or drink from the website of a restaurant, takeaway or café; and 54% had ordered from an online ordering and delivery company (e.g. Just Eat, Deliveroo, Uber Eats)—19% using these delivery apps once a week or more often. These represent potentially valuable food environment intervention points.

Since 2020, other societal changes have impacted on food environments and how the population accesses food. The most notable of these are (i) increases in food prices, with inflation for food and non-alcoholic beverages peaking at 19.2% in 2022 [64,65], and (ii) changes to working patterns, with 41% of working adults in Great Britain hybrid working or working from home in the autumn of 2024 [66]. The shift in work location and the reduction in commute time have had knock-on impacts on locations for shopping and out of home eating, as well as on the types, quality and quantity of food purchased and consumed [67]. Again, these represent intervention points that have had greater prominence since 2020.

Over the past 15 years, dietary change interventions have moved beyond focused individual demand-side measures [68,69]—such as food systems and nutrition education [48,70], labelling [71] and nudges [72]—or on environmental levels (i.e. interventions at the supply-side, such as fiscal policies and marketing restrictions), into more structural systems level interventions [73,74]. We argue for the need to take an approach that recognizes the complex socio-economic-ecological interactions that take place between people and their food environment as part of daily life, and that are crucial in shaping what people eat (part of a more holistic systems-based approach) [75]. Transforming food environments provides an opportunity to address the ‘missing middle’, by changing the sourcing, ingredients, recipes and other available choices in the supply chain from ‘fork-to-farm’ [42] rather than focusing on the choice of individual consumers at the stage of food acquisition.

Transforming food environments requires a multi-level approach that addresses at-home, in-store, neighbourhood, local-area, regional and national challenges [57,76]. Additionally, it influences associated behaviours and practices of provisioning, shopping, storing, preparing [77], cooking and waste management. Likewise, we stress that the actions below, which enable access to healthy and sustainable food, need to be paired with actions to address availability, affordability and desirability in order to be fully effective [78]. We also stress that individual circumstances still matter—with the variety of lived experience [79] and demography, geography, income, etc., meaning that each individual in a community faces different barriers, enablers and facilitators [80]. Therefore, when developing interventions based upon the actions below, taking into account stakeholder views is crucial [81]. Finally, we acknowledge a fundamental challenge: the food system is a commercial entity governed by economic rules. To leverage pro-social change, we must engage with these economic dynamics.

From the TUKFS research we find three common food environment actions suggested across multiple projects. Firstly, to *scale up supermarket-based interventions: such as promoting healthier options and reducing the visibility of unhealthy foods*, can significantly influence consumer choices [82–84]. This can also include featuring ‘missing middle’ food access interventions, discussed further below, and be used to help reduce climate impacts. Secondly, to *expand voucher schemes to subsidize costs, making healthy and sustainable food more affordable and accessible to low-income households*, encouraging better dietary habits [85,86]. Vouchers can go beyond households and into schools through auto-enrolment in school meals [87–93], and similar schemes. And finally, to *standardize and simplify health and environmental labelling* to allow consumers to rapidly view and process labelling information ‘at a glance’, to make informed choices when purchasing healthy and sustainable foods [71,94,95].

We propose the following three ‘missing middle’ structural actions to enhance the food environment approach. Firstly, to *change portion sizes and packaging, to edit the choice architecture in supermarkets and reduce calorie consumption* [96–98], *e.g. providing and normalizing smaller portion sizes to align with dietary advice* has positive health and environmental sustainability outcomes, which has knock-on effects for product selection (the amount of product purchased per shopping event) as well as household food waste [97,98], thus reducing climate impacts. Secondly to *incentivize rollout of fortified* [99–103]*, biofortified* [104] *and healthier genetic variants for* [105] *bread and other products*: modifying the default bread product to be healthier could have wider health outcomes due to the scale of bread consumption. For example, this can be done by exploiting the genetic variation in wheat and using varieties with higher fibre [105]. And finally, to *change recipes and menus in hospitality and wider public sector catering* (hospitals, schools, prisons, military, etc.) to reduce high climate impact ingredients and increase healthy and sustainable options—such as fruits and vegetables [106–108]. Simply strategically swapping dishes on a weekly menu is in principle sufficient to generate meaningful reductions in carbon footprint and saturated fatty acid intake, without impacting consumer satisfaction [109,110].

**Summary of action areas on Theme C: transforming food environments for healthy and sustainable food**

C1. Scale up supermarket-based interventions such as promoting healthier options and reducing the visibility of unhealthy foods [82–84]. Action: Supermarkets and government.

C2. Expand voucher schemes to subsidize costs, making healthy and sustainable food more affordable and accessible to low-income households [85]. Action: Government.

C3. Standardize and simplify health and environmental labelling to allow consumers to rapidly view and process labelling information ‘at a glance’ [71,94]. Action: Government.

C4. Change portion sizes and packaging, to edit the choice architecture in supermarkets and reduce calorie consumption, e.g. providing and normalizing smaller portion sizes to align with dietary advice [96–98]. Action: Manufacturers and supermarkets.

C5. Incentivize rollout of fortified, biofortified and healthier genetic variants for bread and other products [99,100,105]. Action: Defra.

C6. Change recipes and menus in hospitality and wider public sector catering to reduce high impact ingredients and increase healthy sustainable options [109,110]. Action: Caterers.

## 5. Theme D: empowering communities

While much attention is given to individual actions to change food consumption, research has shown the importance of empowering communities to identify actions and create innovations. The term ‘community’ is widely used and encompasses different meanings, ranging from the sense of a geographically defined neighbourhood, to a group of people who share beliefs or experiences, to the sense of togetherness brought about by social connectivity. In this theme we explore the mechanisms by which people can be given agency, choice and the ability to access power and resources that they cannot access as individuals.

Many of the policy instruments governing our food system result in people feeling judged, stigmatized or patronized [12,111]. Research across the TUKFS Programme has consistently shown that people have aspirations to eat a more healthy and sustainable diet, but structural barriers such as lack of resources, money, physical access, time, skills or energy prevent such aspirations being achieved. Community-based social enterprise initiatives are found to empower people to find alternatives and access quality food. Examples include community food hubs and cafes that provide affordable food while also providing training and experience in cooking and preparing healthy food. These initiatives are rooted in their communities so they can develop public health strategies starting from local understandings, rather than preaching or stigmatizing [112]. Transformation can come from working with organizations already embedded in their communities and where there is social innovation based on addressing community need rather than maximizing shareholder value. We suggest *supporting robust social enterprise business models through inspiring start-ups with advice, mentoring and finance to support their scaling up* [113]. This requires a range of support organizations and an enabling environment created by local authorities.

Empowerment of particular communities of producers can lead to an evolution of producer-centric localized food systems which enable farmers and producers involved in processing to add value to their raw materials and sell more directly to end consumers. This can result in regenerative approaches to farming being rewarded with premiums, in recognition of the ecosystems services they are providing alongside the provision of healthy food ([114] and references in §2). We suggest *rebalancing the power within the supply chain towards food producers, to ensure they receive prices that cover the costs of transitions to regenerative approaches and are rewarded for ecosystem services*. This can be achieved through educating consumers, cooperation among small producers and ethical sourcing standards. This should be supported by regulatory action driven from central government to demand better prices from corporate buyers, local markets and public procurement. Public funding from Defra for environmental stewardship is also supporting farmers moving to regenerative approaches.

Research that uses co-production approaches gives a voice to the marginalized through ‘doing with’, rather than ‘doing to’ or ‘doing for’. This co-production is inherently messy and complex to manage, leading many researchers and policymakers to shy away from embracing its methods. Co-production values the lived experience of all actors seeking to transform the food system, particularly those in rural and urban communities whose voices are seldom heard. However, TUKFS projects have demonstrated how shared ideals and practical solutions for food systems transformation are enabled by co-production [113,115]. Approaches such as community researchers and participatory research aim to manage power dynamics to ensure equitable but realistic decision-making and support inclusivity [115–117]. This requires approaches to transdisciplinary work that accepts that there are tensions in food systems but engages diverse parts of communities, invests in building relationships and builds capabilities of those being excluded to participate in research and decision-making [118]. We suggest that *co-production become the default process for research and decision-making that impacts on communities*.

**Summary of action areas on Theme D: empowering communities**

D1. Support robust social enterprise business models through inspiring start-ups with advice and mentoring and providing finance to support their scaling up [112,113]. Action: Foundations and civil society organizations providing business support, banks and other investors, Department for Business and Trade, Department of Culture, Media and Sport.

D2. Rebalance the power within the supply chain towards food producers, to ensure they receive prices that cover the costs of transitions to regenerative approaches and are rewarded for ecosystem services [92,114]. Action: Unions and associations representing farmers and producers, civil society organizations supporting certification systems, responsible businesses supporting their supply chains, Defra.

D3. Make co-production the default process for decision-making that impacts on communities [112,113,115–117]. Action: Local government, Defra, UK Research and Innovation, civil society organizations giving a voice to the excluded.

## 6. Theme E: transforming policy and governance

Policies have been described as important levers for food system change [119]. There are many different activities and outcomes related to food which must be targeted by policymakers wishing to transform national and local food systems. Many actions to transform the food system are either directly or indirectly initiated by policy [120]. At present only a small number of possible policy levers in the toolbox are being applied to do this [119]. A much wider range of innovative policies and practices, delivered in concert, must be implemented if transformation is the aim [121,122].

Better policies are needed [28,119,123–125]; policies that are more effective at delivering coherently on the daunting environmental, social and economic triple sustainability challenge represented by food systems [28]. This includes increasing the use of more robust and effective measures, such as legally binding targets for human and planetary health [119,123–125]. Better policy also means redesigning *existing* policies to improve their effectiveness. We suggest *applying a systems approach, which situates a policy measure within a wider policy mix, and identifies its impacts across a range of food system outcomes*, as one way to address effectiveness. A powerful example, which cuts across much of TUKFS, is public sector food.

Public food procurement provides 5.5% of all food sales in the UK [124], thereby providing a significant lever to support sustainable food production and consumption, as demonstrated in Scandinavian countries [22,126–129]. Policy can enable public sector catering shifts towards regenerative agriculture (§2), increases in plant proteins such as beans (§3), or changes in the food environment (§4). Some pockets of good policy and practice exist in relation to public sector catering. But beyond these, barriers—including weak standards and poor monitoring, and a piecemeal approach which fails to connect procurement to available supply, and nutrition to sustainability [91,129,130]—dampen the transformative potential of public sector food policy [119]. We propose that *both national and local policies related to public sector procurement are considered for their food systems impacts*, and designed to more effectively support goals such as the transition to regenerative agriculture and improving the health of the nation.

We suggest *delivering more effective policy by improving links between national and local actions*. An example of this is policy on free school meals. Currently, 11% of children entitled to free school meals do not take them up, but welfare data can be used to automatically register them, and parents can be given the option to opt-out. This has now been demonstrated to be effective in a number of local authorities, enabling thousands of children to access free meals [93].^3^ Access to school meals could further be increased by extending free school meal provision to higher income levels, or universally, or by subsidies. Four options were investigated for their cost-effectiveness, taking into account healthcare and productivity—all were found to provide a positive return on investment over a 20 year period [132], with subsidized school meals offering the highest cost–benefit ratio. Inconsistencies in school food quality can be addressed with mandatory and enforced food standards [22]. Improved coherence between national and local levels of policymaking—through better data sharing, communication, analysis of enablers and barriers, and more formal spaces for knowledge sharing between different government levels [133,134]—could ensure such opportunities to maximize effectiveness are capitalized on.

Introducing and improving individual policies is only part of the solution. Better policy also means designing, implementing and evaluating policies and practices in the context of the wider food system, to ensure that actions reinforce, rather than undermine, one another [28,119,129,135]. We suggest that *improved understanding of a local food system, including through the application of systems thinking and synthesis of data*, can support a holistic approach, which supports policy coherence [135–139], and helps manage complexity when deciding where and how to intervene [24,140–142]. Again, there are some examples of where understanding local food systems has been prioritized and assigned dedicated resources, for example in London [53,143] and Sheffield [144–146], but local actors—government, practitioners—require further support [147], including resources, and from the research community, and for some data types also the private sector—to fully understand their context and make informed and effective decisions.

Better policymaking *processes* and governance structures are also required. While evidence on the most effective processes and structures is yet to be produced, there continues to be widespread agreement that the current approach is not fit for purpose [119,122,125,148]: a (future) transformed food system demands a transition from the current reactive, disjointed, non-transparent mode of governance to a proactive, holistic, long-term-focused one [24]. It has become trite to observe that this demands better integration of government departments with a role in influencing food systems [124,149–152], and more inclusive and representative involvement of all food system actors and interests. Nevertheless such recommendations remain top of the list of priorities for those working on food systems, and are likely to remain so in lieu of any implemented mechanisms to support connected policy.

More integrated and inclusive ways of working can be enabled by procedural tools such as integrated, holistic food strategies; cross-cutting bodies; and formal mechanisms to involve stakeholders outside government—including citizens—in policymaking. The local, sub-national level has been established as an important transition space for such new food governance structures and processes [143,147,153,154]. Development, implementation and monitoring of local food plans, or strategies, which draw together a range of food system interests and actions, has been a TUKFS Programme focus in Yorkshire [24], North Yorkshire, York, Sheffield [144,145], Leicestershire [141,142] and Birmingham [147]. This has involved building the evidence base on how cross-cutting bodies such as Local Food Partnerships (LFPs), which bring together the public, private and community sectors, help deliver a food systems approach. Work with partnerships in Birmingham, Bristol, Rotherham and Sheffield, and North Yorkshire highlights good practice and explores how this might be scaled up. These findings support the positive value placed on LFPs in the National Food Strategy Independent Review (NFSIR). Yet evidence also highlights how fragile bodies such as LFPs are relying on short-term funding and volunteers. We suggest *longer term support for Local Food Partnerships from national government, and funding* [147,155]*, along with a new focus on food policy models at the regional level* where new forms of governance structures linked to devolution are emerging, e.g. Mayoral Combined Authorities, which TUKFS projects are exploring with the FixOurFood Commission in Yorkshire [155].

Local-level governance has been the TUKFS Programme’s primary research focus. But its coincidence with the development of several major food systems strategies in England (the NFSIR, subsequent Government Food Strategy, and 2025 Food Strategy) along with a Parliamentary Select Committee Inquiry into Food, Diet and Obesity, involving TUKFS experts, means that national food policymaking is also a focus of TUKFS Programme suggestions. These include proposals for a more ambitious, long-term and systemic food strategy. We encourage the *formation of a dedicated food systems cross-government body as a governance mechanism for bringing considerations of population and planetary health together* [124,125,151,152,156], and protecting against the fragility of food strategy initiatives. The benefits and limitations of situating such a body within or at arm’s length from government would be further investigated—for example, evaluating the ability to pressure departments into action versus independence and protection from political cycles [156]. Due to previous food policy bodies not fulfilling their potential [156,157], more embedded legislative mechanisms—such as the Good Food Nation Act in Scotland—are likely to be required, and *recommendations for a Food Act, as outlined in the National Food Strategy Independent Review* [124] *should be also revisited*. The recent introduction of a Food Strategy Advisory Board [158], described as bringing together ‘senior leaders from across the food system’ is a promising development, though it is understood that this body is convened to advise on a discrete policy project, rather than as a permanent institution with longevity and scrutiny powers built in. It is also questionable whether it represents wider food system interests, given the dominance of post-farmgate food sector actors and poor representation of planetary health experts.

We suggest the *better inclusion of food system actors, from farm to fork, including citizens*, which is required for a more systemic approach to policy and governance, *with consideration of a formal mechanism for participation, recognizing the range of actors and perspectives involved* in the food system. Many of the TUKFS Programme’s projects, which are designed around co-production principles, have confirmed there is an appetite for involvement in policy discussions and design, from groups ranging from citizens [81,88,159,160], to civil society organizations [112,161], to farmers (see §2). Because systems change requires collaborative action to be taken beyond public policy, the same principle applies to involving citizens in the co-production of actions with the private sector, such as retail businesses (see §5).

Formal mechanisms—such as public dialogues [124,162] or conversations [163], or a body such as a ‘Citizens Forum’ [150]—can support policy participation, although evidence on their impact is limited. The formal citizen involvement in the development of the UK Government Food Strategy [164] is a promising development, though it is not clear if this will be a time-limited or permanent mechanism. There are examples from countries such as Brazil of permanent bodies which can facilitate participation [156]. Evidence from the TUKFS Programme also confirms that co-production is not a panacea: limits exist in effectiveness and credibility [162]. There are challenges, for instance, associated with incorporating a diversity of voices—particularly those which are underrepresented and excluded—when addressing a complex and value-laden subject such as food [28,146,162]. It is important to recognize the range of perspectives and values related to food, rather than present a coherent singular ‘public’, and to apply best practice on public engagement [165]. Participation in policymaking is not always positive, and raises the spectre of corporate interests and influence [125].

The way we evaluate policies, interventions and transformations in food systems is a key component of governance. Evaluating transformation means transforming conventional approaches to evaluation, to appreciate evaluands (e.g. a transition initiative or food system) as unique, entangled, nested, dynamic and uncertain systems [166]. Such evaluation would also employ more diverse, developmental, contextually adapted and future-sensitive evaluation approaches; promote justice; embrace diverse and marginalized perspectives; and reduce evaluator–evaluand polarization by shifting power towards evaluation users. It would do all of this while also fostering a more autonomous evaluation profession, and mutualistic partnerships of knowledge and action. Most fundamentally, it would recentre values, reflexivity and learning at the heart of evaluation practice, and ensure that the core purpose of evaluation is to support systemic, societal transformation towards regenerative futures [166]. We suggest *adopting more developmental, collaborative and transformation-focused approaches to evaluation and embedding these across food system governance*, seeing evaluation as part of change processes rather than separate from them [166].

Finally, better food systems policy is reliant on better research evidence. The current evidence ecosystem is diverse and fragmented, with different activities and outcomes addressed in isolation, rather than in their wider food system context, and with a lack of synthesis into policy-ready knowledge [167–170]. There is also a need to incorporate more diverse forms of knowledge, as noted above in relation to participatory governance. The discussions that take place both within and outside academia would benefit from *bringing evidence together in an evidence centre, which includes an open access repository for food papers and other outputs*, to support policymakers to take a systemic approach [121,124,170].

**Summary of action areas on Theme E: transforming policy and governance**

E1. Apply a systems approach to policy design—for example, ensuring both national and local policies related to public sector procurement are considered for a range of food system impacts across health, environment and economy [22,126]. Action: All relevant national, regional and local government departments; research evidence generators.

E2. Deliver more effective policy through improving links between national and local actions [133,134]—e.g. access to free school meal provision could be improved through national auto-enrolment of pupils eligible for free school meals and expansion of provision [93,132]. Action: All relevant national, regional and local government departments; research evidence generators, practitioners including civil society organizations.

E3. Improve the understanding of a food system context where interventions will be made, through the application of systems thinking and synthesis of data [24,139,141,142]. Action: Education actors and courses to build food systems thinking capacity for practitioners across the sector, e.g. TUKFS Centre for Doctoral Training [171].

E4. Implement longer term support for Local Food Partnerships from national government, and funding [147,155], along with a new focus on food policy models at the regional level [155]. Action: National, regional and local governments; civil society organizations supporting food partnership development, and leading food-related organizations; research funders (research on regional food policy).

E5. Form a dedicated food systems cross-government body as a governance mechanism for bringing considerations of population and planetary health together [124,151,152,156]. Action: National government.

E6. Revisit the recommendations for a Food Act, as outlined in the National Food Strategy Independent Review [124]. Action: National government, research evidence generators.

E7. Improve inclusion of food system actors, from farm to fork, including citizens [81,88,159,160] with consideration of a formal mechanism for participation, recognizing the range of actors and perspectives involved. Action: National, regional and local governments; practitioners (public sector professionals, private sector, civil society); research evidence generators.

E8. Adopt more developmental, collaborative and transformation-focused approaches to evaluation, and embed these across food system governance [166]. Action: National, regional and local governments; research evidence generators, including evaluators, research funders of evaluations, education actors and courses to build food systems thinking capacity for practitioners across the sector e.g. UKFS Centre for Doctoral Training [171].

E9. Bring evidence together in an evidence centre, drawing on lessons from the What Works Network, and incorporating an open access repository for food papers and other outputs [121,170]. Action: National government, research funders, research evidence generators.

## 7. Discussion

The UK food system is a complex socio-technical system, involving large numbers of the population from primary producers, through manufacturing, transport and logistics, retail and hospitality—with all citizens, as consumers, having an opinion on food. This is consistent with many of the action areas highlighting the need for co-development with food system actors and with communities, of both transformative action areas (D3, E7) and messaging (A1). The projects of the TUKFS Programme have demonstrated this co-production approach in their research, working with and elevating less-heard voices. Taking this approach right across food system decision-making is more challenging given the diversity and disconnectedness of the large number of people involved.

The geographically distributed nature of the food system and cultural variations mean that there is no one-size-fits-all solution. Place-based approaches such as Local Food Partnerships are therefore needed to co-produce regionally appropriate solutions; however, these need to communicate with each other to share best practice and be supported centrally to ensure policy coherence across scales (B3, E3, E4).

The need for change comes at a time when food producers are facing financial pressures and uncertainties, with farmers operating at marginal gains or at a loss, while shouldering much of the risk from increasing extreme weather events. Meanwhile, geopolitical instability threatens sourcing of inputs and ingredients and increasing prices, large food manufacturers and retailers are demonstrating significant economic growth [172], and citizens face a cost-of-living crisis. Structural changes and increased support can enable businesses to make decisions more aligned with a transformed system, through advice and financial support for farmers (A2) and businesses (D1, D2), or enabling the redistribution of surplus food to those in need (B4, B5, B6).

There is a role for innovative approaches and products in the future transformed food system, including increasing UK production and consumption of pulses using new bean varieties (B2) and healthier varieties of wheat into staple products such as bread (C5). The TUKFS Programme investigated how these might be carried out in practice, exploring alternative routes to market; however, in order to effectively scale these up to their full potential benefit, the currently unaccounted health and social benefits would need to be taken into account, as discussed in the context of controlled environment agriculture (B1).

The food environment encountered by consumers can be adapted to amplify the action areas already discussed above. Supermarkets and other retailers have considerable power over purchasing patterns and can wield this power to increase purchasing of products produced in a more regenerative way (C1, C4). Improved labelling can also incentivize producers to change suppliers and reformulate (C3). Caterers also wield power over consumer decision-making, including through the choice and scheduling of available options, which has been found to offer significant health and environmental benefits (C6). In particular, the school-food system offers a major opportunity to provide the most disadvantaged children with a hot meal, and TUKFS projects have demonstrated that this can help many more children than it currently does, and deliver return on investment by rolling it out further (E2). Furthermore, modern voucher schemes can help households across multiple types of outlet without the need for stigmatization at the point of purchase (C2). Finally, public procurement offers an important opportunity for the government to take charge of the food system and have a disproportionately positive effect on small businesses (E1).

Addressing the pressing challenges of the 21st century requires new ways of working and a pace that outstrips the traditional ways of doing research. It will involve actors across scales well beyond traditional discipline boundaries and well beyond academia. The TUKFS Programme has demonstrated a new way of doing research, focusing on action research that takes place within the subject of study, engaging with practitioners in real-time. The timeline of information exchange needs to be shorter than a publication cycle of multiple years to carry out and publish research, to help address the current challenges in a timely manner. At the same time, research needs to take place over time periods longer than a traditional funding cycle to enable understanding of the long-term effects of changes, bringing in the real-world constraints of food system actors (A3).

Projects in the TUKFS Programme have exemplified the combination of research, policy and practice required to address UK strategic objectives. Yet the field of food systems research is hampered by poorly defined boundaries and a traditional publishing format using disparate journals, which makes it extremely challenging to find the latest research even within academia, and publishing paywalls pose a barrier to sharing the information with the very policymakers many academics are wishing to influence. Contrast this with a discipline such as astrophysics, which has no such urgent challenges, where papers are usually deposited in a single open access repository at the time of submission (arXiv). Therefore we propose an open access repository for food systems research (E9).

Actions to change the food system can sometimes seem in opposition to one another—e.g. an apparent trade-off between improving health and environmental outcomes [70]—or can seem to place food system actors in opposition. However, multiple TUKFS projects have demonstrated the potential for food system actors to work together successfully. The sprawling nature of the food system generally, the need for such major changes and the food system’s intersection with multiple government departments necessitates cross-government collaboration (E5); a clear co-produced roadmap for change—we encourage a revisit of the recommendation for a Food Act (E6); and appropriately complexity-informed, collaborative and transformation-focused approaches to evaluation and learning (E8). We therefore welcome the government commitment to developing a new UK food strategy and encourage the consideration of the evidence-based action areas emerging from the TUKFS Programme, of which this article provides a snapshot, as well the large body of existing literature in which this work sits.

The work of the TUKFS Programme is ongoing but it is timely to summarize the wealth of outputs arising from the projects and analysis done thus far. We anticipate further insights from the TUKFS Programme in its final year. In addition, through the individual projects and the Centre for Doctoral Training, the TUKFS Programme has nurtured many early career researchers, developing their ability to work across disciplinary boundaries and carry out action research, thus contributing to the next generation of food thinkers. Meanwhile, at this crucial time for UK food policymaking, we urge the consideration of the suggestions made in this paper, and development by food system stakeholders of detailed actions which can make these suggestions a reality, thus helping to make a step change in transformation of the food system.

## Data Availability

This article has no additional data.

## Footnotes

^1^
In this chapter, we focus on action areas arising from the TUKFS projects, citing only those papers in the summary boxes; however, we note that often other literature exists with similar conclusions.

^2^
For succinctness, we write ‘Defra’ as a shorthand for Defra and the corresponding agencies in the Devolved Administrations.

^3^
We note that while there has been much academic and public support for auto-enrolment, the government cites legal and data protection issues as obstacles to further action [131].
